# Supplementary material for: Allocation of resources among multiple daughter cells
Source: bioRxiv. 2025 May 3:2025.05.02.651883. Preprint. [Version 1] doi: 10.1101/2025.05.02.651883 (PMC12247967; doi:10.1101/2025.05.02.651883)
Supplement: Supplement 1 [file NIHPP2025.05.02.651883v1-supplement-1.pdf]

bioRxiv preprint doi: https://doi.org/10.1101/2025.05.02.651883; this version posted May 3, 2025. The copyright holder for this preprint (which was not certified by peer review) is the author/funder, who has granted bioRxiv a license to display the preprint in perpetuity. It is made available under aCC-BY 4.0 International license.

SUPPLEMENTAL FIGURE CAPTIONS

Supplemental Figure 1: Growth assays of 10-fold serial dilutions of the indicated strains plated on YPD and grown for 48 hours at the indicated temperature. Three independent transformants (Colony 1-3) are shown.

Supplemental Figure 2: Effect of LatB on F-actin. Confocal maximum projection images of *act1*^{V75I} cells treated with 50 µM LatB, 625 nM LatB, or vehicle control (DMSO) and then fixed and stained with Alexa488-phalloidin. Images are inverted to highlight dim actin cables. With 50 µM LatB, no clear F-actin structures are seen. With a low dose of LatB, 625 nM, actin patches are depolarized and faint actin cables are only occasionally visible (red arrow). Scale bar, 5 µm.

bioRxiv preprint doi: https://doi.org/10.1101/2025.05.02.651883; this version posted May 3, 2025. The copyright holder for this preprint (which was not certified by peer review) is the author/funder, who has granted bioRxiv a license to display the preprint in perpetuity. It is made available under aCC-BY 4.0 International license.

Supplemental Figure 1

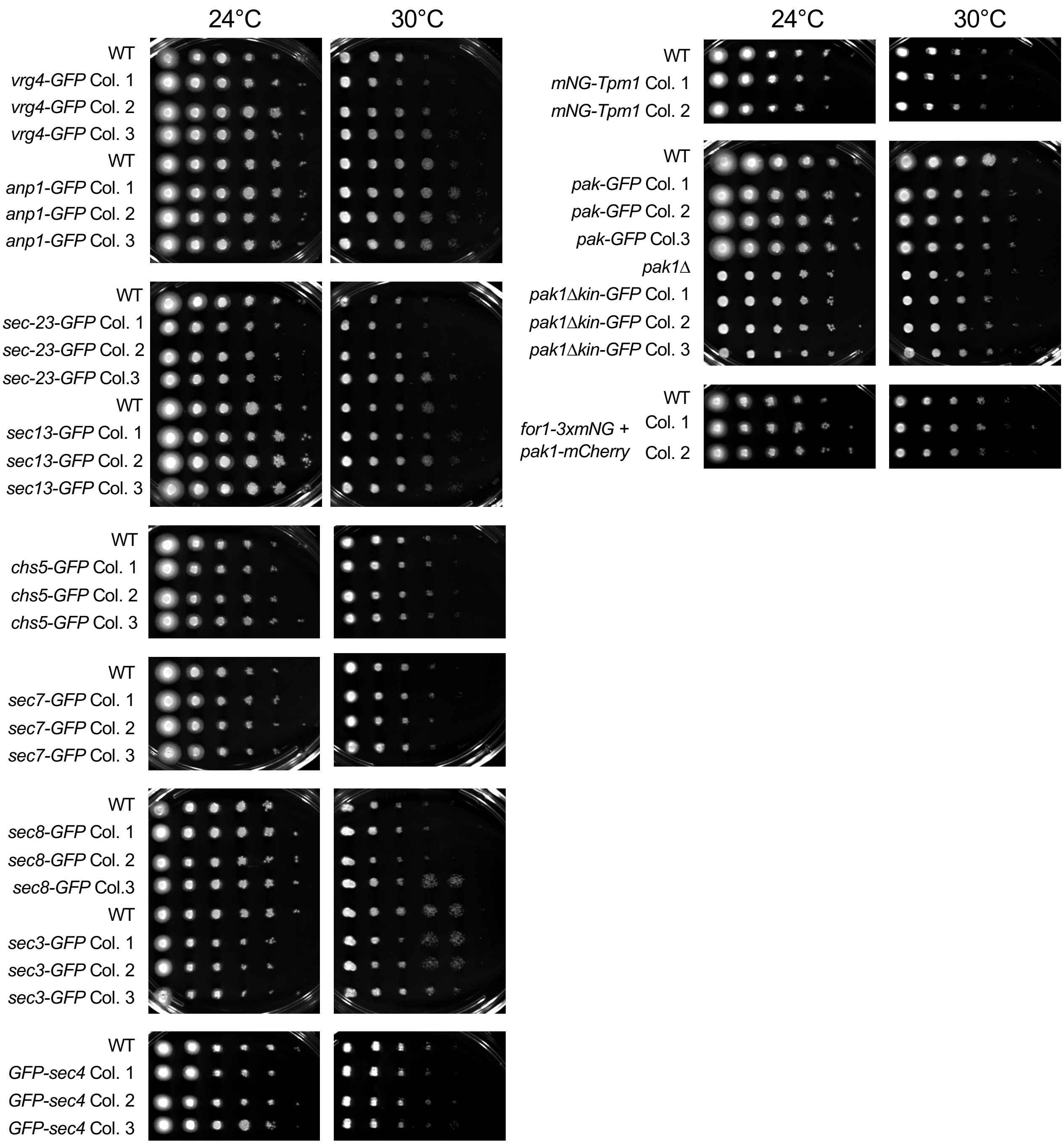

Supplemental Figure 1: Growth assays of 10-fold serial dilutions of the indicated strains plated on YPD and grown for 48 hours at the indicated temperature. Three independent transformants (Colony 1-3) are shown.

bioRxiv preprint doi: https://doi.org/10.1101/2025.05.02.651883; this version posted May 3, 2025. The copyright holder for this preprint (which was not certified by peer review) is the author/funder, who has granted bioRxiv a license to display the preprint in perpetuity. It is made available under aCC-BY 4.0 International license.

Supplemental Figure 2

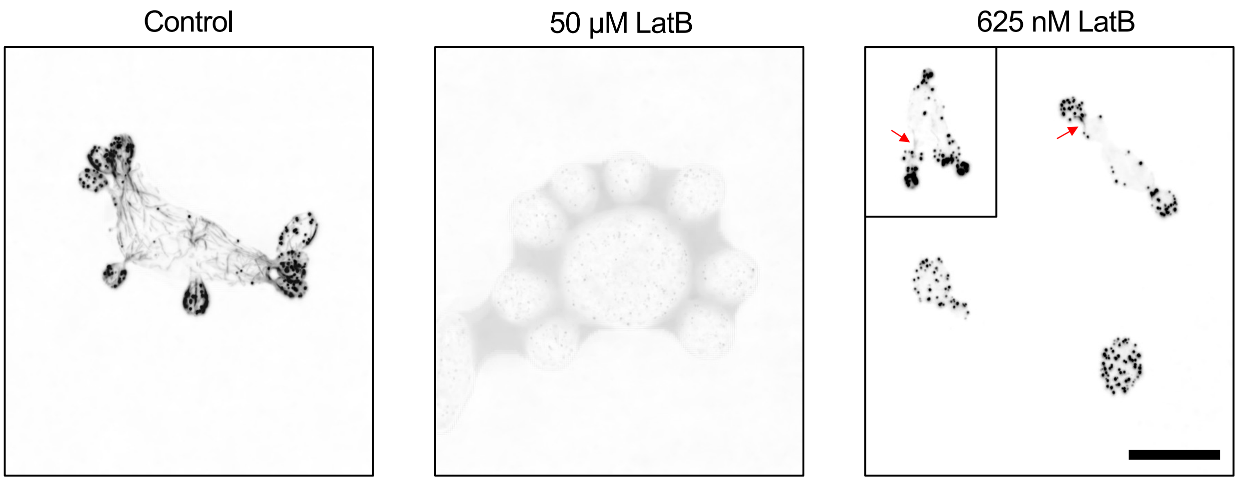

Supplemental Figure 2: Effect of LatB on F-actin. Confocal maximum projection images of *act1*^{V75I} cells treated with 50 μM LatB, 625 nM LatB, or vehicle control (DMSO) and then fixed and stained with Alexa488-phalloidin. Images are inverted to highlight dim actin cables. With 50 μM LatB, no clear F-actin structures are seen. With a low dose of LatB, 625 nM, actin patches are depolarized and faint actin cables are only occasionally visible (red arrow). Scale bar, 5 μm.

bioRxiv preprint doi: https://doi.org/10.1101/2025.05.02.651883; this version posted May 3, 2025. The copyright holder for this preprint (which was not certified by peer review) is the author/funder, who has granted bioRxiv a license to display the preprint in perpetuity. It is made available under aCC-BY 4.0 International license.

Supplemental Table 1

Strain Name	Strain description	Relevant Genotype	Source
DLY23540	WT *A. pullulans*		Gostinčar *et al*., 2014
DLY24919	Strain expressing a cytosolic marker (3xmCherry) integrated at the *URA3* locus	*URA3:spH2Bp-3xmCherry*	This study
DLY25283	Strain expressing a cytosolic marker (3xmCherry) integrated at the URA3 locus and a tropomyosin marker (mNG-Tpm1) integrated as an additional copy	*URA3:3xmCherry; mNG-Tpm1:HYG ^{R}*	This study
DLY24872	Strain expressing the F-actin marker Lifeact-3xmCherry in a background with mutant actin capable of binding phalloidin	*URA3:scACT1p-Lifeact-3xmCherry; act1 ^{V75I}*	Wirshing *et. al*., 2025
DLY24715	Endogenous *SEC13* C-terminally tagged with GFP	*SEC13-GFP:HYG ^{R}*	This study
DLY24712	Endogenous *SEC23* C-terminally tagged with GFP	*SEC23-GFP:HYG ^{R}*	This study
DLY24706	Endogenous *VRG4* C-terminally tagged with GFP	*VRG4-GFP:HYG ^{R}*	This study
DLY24709	Endogenous *ANP1* C-terminally tagged with GFP	*ANP1-GFP:HYG ^{R}*	This study
DLY24938	Endogenous *CHS5* C-terminally tagged with GFP	*CHS5-GFP:HYG ^{R}; URA3:3xmCherry*	This study
DLY24935	Endogenous *SEC7* C-terminally tagged with GFP	*SEC7-GFP:HYG ^{R}; URA3:3xmCherry*	This study
DLY24501	Strain expressing *SEC4* N-terminally tagged with GFP as an additional copy at the *URA3* locus	*URA3:scACT1p-GFP-SEC4*	This study
DLY25370	Strain expressing a cytosolic marker (3xmCherry) integrated at the *URA3* locus and endogenous *SEC8* C-terminally tagged with GFP	*URA3:spH2Bp-3xmCherry; SEC8-GFP:NAT ^{R}*	This study
DLY25394	Strain expressing a cytosolic marker (3xmCherry) integrated at the *URA3* locus and endogenous *EXO70* C-terminally tagged with GFP	*URA3:spH2Bp-3xmCherry; EXO70-GFP:NAT ^{R}*	This study
DLY25367	Strain expressing a cytosolic marker (3xmCherry) integrated at the *URA3* locus and endogenous *SEC3* C-terminally tagged with GFP	*URA3:spH2Bp-3xmCherry; SEC3-GFP:NAT ^{R}*	This study
DLY24853	Strain expressing a nuclear marker (spH2B-mCherry) integrated at the *URA3* locus	*URA3:spH2Bp-spH2B-mCherry*	Petrucco *et. al.*, 2025
DLY25905	Endogenous *PAK1* C-terminally tagged with mCherry and endogenous *FOR1* C-terminally tagged with 3xmNG	*PAK1-mCherry:NAT ^{R}; FOR1-3xmNG:HYG ^{R}*	This study
DLY25647	Strain expressing a cytosolic marker (3xmCherry) integrated at the *URA3* locus and endogenous *PAK1* C-terminally tagged with GFP	*URA3:spH2Bp-3mCherry; PAK1-GFP:NAT ^{R}*	This study
DLY25650	Strain expressing a cytosolic marker (3xmCherry) integrated at the *URA3* locus and endogenous *PAK1* modified to replace the C-terminal kinase domain with GFP	*URA3:spH2Bp-3mCherry; pak1ΔC-GFP:NAT ^{R}*	This study

bioRxiv preprint doi: https://doi.org/10.1101/2025.05.02.651883; this version posted May 3, 2025. The copyright holder for this preprint (which was not certified by peer review) is the author/funder, who has granted bioRxiv a license to display the preprint in perpetuity. It is made available under aCC-BY 4.0 International license.

Supplemental Table 2

Plasmid Name	Relevent genes	Selectable marker	Plasmid description
DLB4820	*3xmCherry*	*URA3*	Plasmid for integration of 3xmCherry at the native *URA3* locus
DLB4897	*mNG-TPM1*	*HYG* ^{R}	Plasmid for expression of N-terminally tagged TPM1 under control of the native TPM1 promoter and terminator
DLB4847	*spH2B-mCherry*	*URA3*	Plasmid for integration of the Chytrid *S. punctatus* histone H2B tagged with mCherry at the native *URA3* locus
DLB4775	*GFP-SEC4*	*URA3*	Plasmid for integration of *GFP-SEC4* at the native *URA3* locus
DLB4992	*3xmNG*	*HYG* ^{R}	pAPInt series plasmid for 3-part-PCR integration of a C-terminal 3xmNG tag with Hyg. Selection

bioRxiv preprint doi: https://doi.org/10.1101/2025.05.02.651883; this version posted May 3, 2025. The copyright holder for this preprint (which was not certified by peer review) is the author/funder, who has granted bioRxiv a license to display the preprint in perpetuity. It is made available under aCC-BY 4.0 International license.

Supplemental Table 3

Primer name	Sequence	Relevant gene	Protein ID# (MycoCosm)	Primer description
Vrg4_ups_F	GTAGTTCGATTCTCATGACCACGACC	*VRG4*	355218	Primers used in 3-part-PCR to tag *VRG4*
Vrg4_ups_R	CATAGAACCAGAACCAGCACCGTCACCTGACTTGAAGCTGTCCTTCATGCTC			
Vrg4_pAPint_F	AGCAGCCAGAGCATGAAGGACAGCTTCAAGTCAGGTGACGGTGCTGGTTCTGG			
Vrg4_pAPint_R	GATCATGGAAAACTTGCTCTCGTGTCTGATCGCATAGGCCACTAGTGGATCTG			
Vrg4_dwn_F	TATACTGCAGATCCACTAGTGGCCTATGCGATCAGACACGAGAGCAAGTTTTCCATG			
Vrg4_dwn_R	CCTTCAACATCCAGAACGACTTCACC			
Anp1_ups_F	AAGCACGCAACTGGCTGTTGAG	*ANP1*	343193	Primers used in 3-part-PCR to tag *ANP1*
Anp1_ups_R	CATAGAACCAGAACCAGCACCGTCACCGATGATGGCCTCCTTCTTTTGCC			
Anp1_pAPint_F	GCGGCAGCCGGGCAAAAGAAGGAGGCCATCATCGGTGACGGTGCTGGTTCTGG			
Anp1_pAPint_R	TTTATACCCATGCACAACTCCCGATATTCCCGCATAGGCCACTAGTGGATCTG			
Anp1_dwn_F	TATACTGCAGATCCACTAGTGGCCTATGCGGGAATATCGGGAGTTGTGCATGGG			
Anp1_dwn_R	GATGGTCTGGCACATGGTGACC			
Sec23_ups_F	CTCGTGGAAGATGTGTGGTATCAACC	*SEC23*	324106	Primers used in 3-part-PCR to tag *SEC23*
Sec23_ups_R	CATAGAACCAGAACCAGCACCGTCACCGCTGTTACCGCTGACGGCCAAC			
Sec23_pAPint_F	CACTTGATGAAGTTGGCCGTCAGCGGTAACAGCGGTGACGGTGCTGGTTCTGG			
Sec23_pAPint_R	TTTCCTCTCTCTCTCTACTGATCGAACACTCGCATAGGCCACTAGTGGATCTG			
Sec23_dwn_F	TATACTGCAGATCCACTAGTGGCCTATGCGAGTGTTCGATCAGTAGAGAGAGAGAGG			
Sec23_dwn_R	AAGTTCTAGTCAGGAGCCTCGATCG			
Sec13_ups_F	GTATGCTGCCAAACACCTCCTGG	*SEC13*	378285	Primers used in 3-part-PCR to tag *SEC13*
Sec13_ups_R	CATAGAACCAGAACCAGCACCGTCACCCTCCTCGATGGTCTTGACGCAGTC			
Sec13_pAPint_F	GGAGGATGGGACTGCGTCAAGACCATCGAGGAGGGTGACGGTGCTGGTTCTGG			
Sec13_pAPint_R	AGTAGCCTCCAGGCTAGGCAGAACAAATTGCGCATAGGCCACTAGTGGATCTG			
Sec13_dwn_F	TATACTGCAGATCCACTAGTGGCCTATGCGCAATTTGTTCTGCCTAGCCTGGAGG			
Sec13_dwn_R	GACAAGACCTGGATAGCTGTGTGC			
Sec7_ups_F	ACAGCTCTCTTCTCTGCAGCG	*SEC7*	269067	Primers used in 3-part-PCR to tag *SEC7*
Sec7_ups_R	CATAGAACCAGAACCAGCACCGTCACCACGACTGCCTCTGCTCGAAC			
Sec7_pAPint_F	TTCAGCGGGCGTCGTTCGAGCAGAGGCAGTCGTGGTGACGGTGCTGGTTCTGG			
Sec7_pAPint_R	GTCCCAACGCTTCTTAACCAAACCTTCACTCGCATAGGCCACTAGTGGATCTG			
Sec7_dwn_F	TATACTGCAGATCCACTAGTGGCCTATGCGAGTGAAGGTTTGGTTAAGAAGCGTTGG			
Sec7_Dwn_R	GTAACCATTGTCCACCTTCCTTGC			
Chs5_ups_F	GCTGCGTAGTCTGAGCCTGTAC	*CHS5*	352483	Primers used in 3-part-PCR to tag *CHS5*
Chs5_ups_R	CATAGAACCAGAACCAGCACCGTCACCCAGAGCCACATCATCAAATTCGGTCG			
Chs5_pAPint_F	AAGGCACCGACCGAATTTGATGATGTGGCTCTGGGTGACGGTGCTGGTTCTGG			
Chs5_pAPint_R	GAACGCTAGTCACAAATTACGGTCCTCATTCGCATAGGCCACTAGTGGATCTG			
Chs5_dwn_F	TATACTGCAGATCCACTAGTGGCCTATGCGAATGAGGACCGTAATTTGTGACTAGCG			
Chs5_Dwn_R	GCTCCAAGGAGCAAATCCTGC			
Pak1_ups_F	GCATGTCAAGCATGACTGATGCTCAGG	*PAK1*	374035	Primers used in 3-part-PCR to tag *PAK1*
Pak1_ups_R	CATAGAACCAGAACCAGCACCGTCACCGTGGCCGCTCTGCTTTCTAAATCTCAGC			
Pak1_pAPint_F	GCTCGCCGAGCTGCTGAGATTTAGAAAGCAGAGCGGCCACGGTGACGGTGCTGGTTCTGG			
Pak1-DEL_pAPint_R	TGAGTATATGCAAAGTTTGATGCGAATCGTCACGCCGCATAGGCCACTAGTGGATCTG			
Pak1_dwn_F	TCGCTATACTGCAGATCCACTAGTGGCCTATGCGGCGTGACGATTCGCATCAAACTTTGC			
Pak1_dwn_R	CGACCGTATTGTTGTCTGCCTCC			
Pak1_ups_delK_F	CAAGTTCGAGACCGACAACGACG	*PAK1*	374035	Primers used in 3-part-PCR to replace the kinase domain of *PAK1* with GFP
Pak1_ups_delK_R	CATAGAACCAGAACCAGCACCGTCACCGGAGTCCAAAGGACGCTCCTTGG			
Pak1_delK_pAPint_F	GCGAGGTCGTCTCCAAGGAGCGTCCTTTGGACTCCGGTGACGGTGCTGGTTCTGG			
Pak1-DEL_pAPint_R	TGAGTATATGCAAAGTTTGATGCGAATCGTCACGCCGCATAGGCCACTAGTGGATCTG			
Pak1_dwn_F	TCGCTATACTGCAGATCCACTAGTGGCCTATGCGGCGTGACGATTCGCATCAAACTTTGC			
Pak1_dwn_R	CGACCGTATTGTTGTCTGCCTCC			
apFormin_ups_F	TCTCGGTGAGATGAAGCGGAC	*FOR1*	369820	Primers used in 3-part-PCR to tag *FOR1*
apFormin_ups_R	CCCTTAGACATAGAACCAGAACCAGCACCGTCACCAGATAACCTGGGACTGCCAAGTG			
apFormin_pAPInt_F	ATTTCAGCACACCACTTGGCAGTCCCAGGTTATCTGGTGACGGTGCTGGTTCTGG			
apFormin_pAPInt_R	GTGACCCAAACAGCTTTCCTATCCTACTCTAGTGCCGCATAGGCCACTAGTGG			
apFormin_dwn_F	GTCGCTATACTGCAGATCCACTAGTGGCCTATGCGGCACTAGAGTAGGATAGGAAAGCTG			
apFormin_dwn_R	GTCTACCTCTCCTTCCTCCGCAG			
apEXO70_ups_F	CGACGCTGAAAGACCTTGATCGTC	*EX070*	346362	Primers used in 3-part-PCR to tag *EXP70*
apEXO70_ups_R	AGACATAGAACCAGAACCAGCACCGTCACCCCCCAAATTCGAGAGCACCGC			
apEXO70_pAPint_F	CAACTCAATGCGGTGCTCTCGAATTTGGGGGGTGACGGTGCTGGTTCTGG			
apEXO70_pAPint_R	CCACGCTAAAGTCCAAAACCCCATCTTCCTCGCATAGGCCACTAGTGGATCTG			
apEXO70_dwn_F	TATACTGCAGATCCACTAGTGGCCTATGCGAGGAAGATGGGGTTTTGGACTTTAGC			
apEXO70_dwn_R	ACGACCTTTGCATCAGCATCATTC			
apSEC8_ups_F	GCAAATCGACGCCACTGCTGAG	*SEC8*	378094	Primers used in 3-part-PCR to tag *SEC8*
apSEC8_ups_R	AGACATAGAACCAGAACCAGCACCGTCACCATCCAAACCCCTAAGCGCACTTTC			
apSEC8_pAPint_F	GAAGTGGAAAGTGCGCTTAGGGGTTTGGATGGTGACGGTGCTGGTTCTGG			
apSEC8_pAPint_R	AACACACACAAATCCAACAAACTCCCCCCACGCATAGGCCACTAGTGGATCTG			
apSEC8_dwn_F	TATACTGCAGATCCACTAGTGGCCTATGCGTGGGGGGAGTTTGTTGGATTTGTG			
apSEC8_dwn_R	AAGACAATGCCGACCCAATGGAC			
apSEC3_ups_F	TGAATTGAGAACGCTTATGGAGTGG	*SEC3*	402877	Primers used in 3-part-PCR to tag *SEC3*
apSEC3_ups_R	AGACATAGAACCAGAACCAGCACCGTCACCAATCCTCTTGAATCCTTCTTTTGTATCTTC			
apSEC3_pAPint_F	GAAGATACAAAAGAAGGATTCAAGAGGATTGGTGACGGTGCTGGTTCTGG			
apSEC3_pAPint_R	TTCATAGCTGTCATTTTTCGTTTCTCAAACCGCATAGGCCACTAGTGGATCTG			
apSEC3_dwn_F	TATACTGCAGATCCACTAGTGGCCTATGCGGTTTGAGAAACGAAAAATGACAGCTATG			
apSEC3_dwn_R	CAGCACCTACGATGAAGGTCAAC			
